# Comparative evaluation of four household food‑waste composting systems for decentralized circular waste management

**DOI:** 10.1007/s11356-026-38301-x

**Published:** 2026-10-10

**Authors:** Stanislava Voběrková, Eva Jansová, Martina Vršanská, Tomáš Do, Lenka Vaňková, Petra Martínez Barroso, Ivo Sedláček, Aleš Knoll, Ester Kovaříková, Magdalena Dária Vaverková

**Affiliations:** 1 Department of Chemistry and Biochemistry, Mendel University in Brno, Zemědělská 1, Brno, 613 00 Czech Republic; 2 Department of Applied and Landscape Ecology, Faculty of AgriSciences, Mendel University in Brno, Zemědělská 1, Brno, 613 00 Czech Republic; 3 Department of Experimental Biology, Czech Collection of Microorganisms, Faculty of Science, Masaryk University, Kamenice 5, Brno, 625 00 Czech Republic; 4 Department of Animal Morphology, Physiology and Genetics, Faculty of AgriSciences, Mendel University in Brno, Zemědělská 1, Brno, 613 00 Czech Republic; 5 Faculty of Civil Engineering, Environmental Engineering and Architecture, Warsaw University of Life Sciences (SGGW), Nowoursynowska 159, Warsaw, 02 776 Poland

**Keywords:** Household food waste composting, Composting devices, Compost quality, Microbiological safety, Sustainable waste management

## Abstract

The study evaluated four household food waste composting systems using replicated trials (*n* = 3 per device) with standardized feedstock loads to compare temperature dynamics, physicochemical parameters, compost maturity, and microbiological safety. Although electric units automated mixing and heating, they did not achieve faster overall stabilization, and all devices produced semistabilized material requiring extended curing. Economic and environmental assessments indicated trade‑offs between operational costs, energy use, and waste diversion potential. Screening of culturable isolates did not reveal antibiotic‑resistant strains, though this does not exclude their presence in uncultured microbial fractions. The results highlight the need for additional maturation steps and context‑specific adoption strategies for decentralized circular waste management.

**Supplementary information:**

The online version contains supplementary material available at https://doi.org/10.1007/s11356-026-38301-x.

## Introduction

The increase in global waste production is largely due to the process of urbanization and the continuing growth of the human population (Meybeck et al. 2011). To address these concerns, the adoption of effective waste management strategies becomes imperative. The increasing environmental pressures caused by waste disposal and the scarcity of natural resources belong to the main drivers for the adoption of a circular economy (Bourdin and Jacquet 2025; Sasso et al. 2025), which focuses on the valorization, minimization, and recycling of waste. Europe has made significant progress in transforming waste into a resource, thus promoting sustainable waste management practices such as recycling (Badgett and Milbrandt 2021). A target to recycle 65% of municipal waste by 2035 is included in the European Union’s (EU) revised action plan (“Closing the loop”) (COM 2015).

Among the various waste treatment methods available, composting is emerging as a widely used technique due to its myriad benefits, such as waste sanitization, cost efficiency, and the conversion of waste into a value-added product (Pérez et al. 2023).

Although composting is widely promoted as an accessible and sustainable method for valorizing food waste, its practical implementation at the household scale is accompanied by several well-documented limitations. Small and heterogeneous batches of food waste often fail to reach or sustain thermophilic temperatures, which reduces sanitization efficiency and slows the overall degradation process (Tibu et al. 2019). In addition, insufficient aeration, moisture imbalance, and seasonal temperature fluctuations can promote anaerobic microenvironments, leading to odor formation, leachate production, and acidification of the substrate (Agapiou et al. 2020; Zhang et al. 2016). These conditions frequently result in immature or phytotoxic compost that requires extended curing periods before safe soil application (Boeykens et al. 2015; Sánchez et al. 2017). The quality of household compost is further influenced by user-dependent factors such as irregular mixing, inconsistent moisture control, and limited process monitoring, which contribute to variability in mineralization and humification (Gómez-Brandón et al. 2008; Voběrková et al. 2020). Recognizing these constraints is essential for evaluating the performance and suitability of different household composting systems within decentralized circular economy strategies.

The effectiveness of composting technologies depends on their ability to rapidly and hygienically convert food waste into mature compost with minimal energy input, odor, and maintenance.

Although household composters are increasingly used, systematic evaluations of their technological performance remain limited (Voběrková et al. 2020; Ji et al. 2023; Enebe and Erasmus 2023). Existing studies rarely address how differences in insulation, mixing mechanisms, chamber volume, or automation influence process stability, compost maturity, and hygienization performance, despite evidence that these design parameters substantially affect temperature development, aeration efficiency, and mineralization dynamics (Gómez-Brandón et al. 2008; Sánchez et al. 2017; Zhang et al. 2016). These gaps highlight the need for comparative assessments of household composters under real‑world conditions.

This study provides a comparative, experimentally grounded assessment of household composting technologies relevant for small‑scale users. The objective of this study is to quantitatively compare four household composting systems with respect to temperature dynamics, physicochemical parameters, compost maturity, and microbiological safety. The four composting devices selected for this study represent the most commonly used household‑scale technologies currently available in Central Europe, where decentralized food waste management has been increasingly promoted within circular economy initiatives (Voběrková et al. 2020; Jakimiuk et al. 2021). These systems—electric shredding units, insulated rotary drums, and nonelectric tumblers—cover the dominant technological categories adopted by households seeking to reduce residual waste and valorize biodegradable materials. Their widespread use reflects regional characteristics, including typical household food waste composition, climatic conditions influencing composting performance, and the growing interest in small‑scale biological treatment options across the Czech Republic and neighboring countries. Incorporating these representative technologies therefore enables a realistic assessment of household composting performance under conditions relevant to Central European circular waste management strategies.

Operational characteristics and economic considerations are discussed conceptually to contextualize the experimental findings. Additionally, the study characterizes the final compost products from household systems, assesses their environmental impacts, and highlights the importance of microbiological safety, including the detection of antibiotic-resistant bacteria.

## Materials and methods

### Food waste

The input raw material composition for composting consisted mainly of FW from a local canteen of the Mendel University in Brno and was supplemented with FW from domestic sources. The raw material composition consisted mainly of boiled rice, boiled potatoes (prepared in different ways), and vegetables (vegetable salads, untreated vegetables). Four FW processing devices were used as part of the research. The feedstock used in this study was designed to reflect commonly occurring fractions of household food waste. It consisted of boiled rice, potatoes, and mixed vegetables collected from the Mendel University canteen and supplemented with domestic food waste (bread, meat, dairy). The selection of these components is consistent with household food waste composition studies reporting vegetables, potatoes and other tubers, and cereal- or starch-based foods among frequently occurring food waste categories. Previous studies on small-scale composting (van Dooren et al. 2019; van der Werf et al. 2020; Voběrková et al. 2020) have likewise used comparable food waste feedstocks, further supporting the suitability of the selected material for household-scale composting experiments.

The differences in feedstock mass among the tested devices resulted from their inherent chamber volume and loading capacity. These differences were retained intentionally, as they reflect the operational constraints of the respective technologies and therefore better represent realistic household use than strictly standardized laboratory loading conditions.

### Tested composting devices and their technological processes

**Fig. 1:**
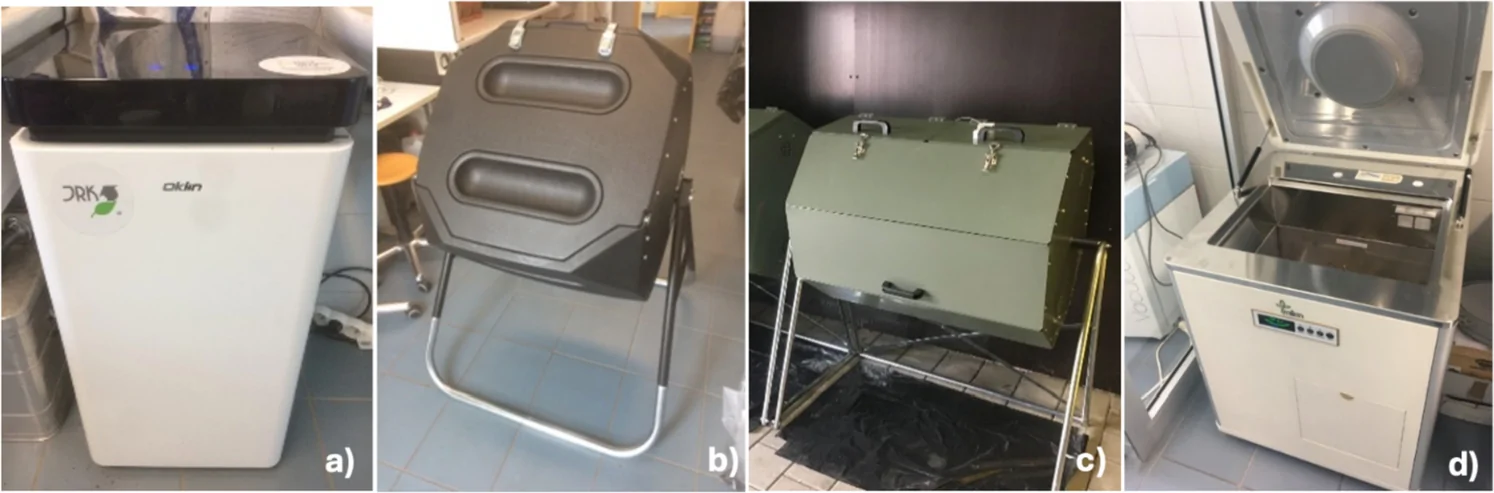
**a** Domestic automatic composter (GG-02). **b** Garden tumbler composter (Lifetime). **c** Double-bin rotating composter (JORA). **d** Automatic FW decomposer (Millen)

Four different devices with different composting time process were tested: household automatic composter (GG-02, Fig. 1a), in which the composting process took 28 days, garden tumbler composter (Lifetime, Fig. 1b) (composting process took 64 days), double-bin rotating composter (JORA, Fig. 1c) (composting process took 103 days), and automatic FW decomposer (Millen, Fig. 1d) (composting process took 83 days). See Supplementary Material (S1) for a detailed description of the composters tested. Insulation plays a pivotal role in maintaining thermophilic temperatures during composting, which are essential for effective pathogen reduction. Differences in insulation among the tested devices may lead to significant temperature fluctuations, affecting the sanitization process and compost maturity. Each composting device was tested in three independent biological replicates (*n* = 3 per device), using identical feedstock composition and analytical procedures.

### Physicochemical analyses of products

The temperature was measured by a manual dial thermometer (Datalogger Termioplus, Poland) incorporated into the composters, and a digital portable thermometer was introduced into the compost for a quick reading. Temperature measurement was repeated three times.

The pH and electrical conductivity (EC) of the aqueous extract of samples were (1:10, w/v, sample/water ratio) measured by a pH electrode (inoLab pH 720, WTW, Czech Republic) and a conductivity electrode (inoLab Cond Level 1, WTW, Czech Republic) according to Vršanská et al. (2024). The result of the measurements was determined at a constant temperature after three repetitions.

Quantitative determination (%) of C and N in final products was performed according to the published procedure Voběrková et al. (2020). Homogenized samples were analyzed in triplicate.

FTIR spectra of humic acids (HAs) were recorded according to Voběrková et al. (2020) using the INVENIO R FTIR Spectrometer equipped with a single-reflection diamond ATR accessory–A225/Q Platinum ATR module (Bruker Optic Inc., Billerica, MA, USA).

The phytotoxicity test was performed using a Phytotoxkit™ by MicroBioTests Inc. The test was carried out in triplicates on samples of the final product where seeds of white mustard (*Sinapis alba*) were tested. The phytotoxicity test monitors the early development of higher plants (precisely seed germination and root growth) exposed to tested soil for at least 72 h in darkness incubated at 25 °C. The calculation of phytotoxicity is founded on a comparison of the number of germinated seeds in tested and reference setup and follows the below formula (1), where *F* can stand even for seed germination index (GI) or root growth index (RI), both used for assessing the phytotoxicity:

1 $$F=\frac{(R-T)}{R\times 100\, (\%)}$$

where *F* is phytotoxicity (%), *R* is the number of germinated seeds in a reference tested sample (pieces) (MicroBioTests Inc.), and *T* is the number of germinated seeds in a tested sample (pieces).

The test procedure followed the standard operational procedure provided by MicroBioTests Inc.

### Microbial analyses of products

Microbiological analysis was performed on the final products. Cultures of *Salmonella* spp., *Escherichia coli*, enterococci, and sporulating bacteria were monitored at the end of the composting process.

#### *Salmonella* spp.*, Escherichia coli, *and* Enterococcus* spp.

For the evaluation of compost hygiene, *Salmonella* spp., *Escherichia coli*, and *Enterococcus* spp. were detected using the method recommended by Czech legislation (ČSN 2020).

The heat-shock method, in which isolates are exposed to a temperature of 80 °C for 15 min, was used to confirm the presence of sporulating bacteria by removing vegetative cells.

After sampling, five samples from the center of each composting device were directly analyzed to detect the presence of bacteria. In the case of *Salmonella*, 25 g of compost was mixed with 225 mL of 0.9% sodium chloride solution (10^–1^ dilution) and shaken at 150 rpm at 4 °C for 1 h. A range of dilutions up to 10^–5^ was prepared in cases of higher colony concentrations. Triplicates of each dilution were used in a series of cultivation steps for analysis.

After preparing a dilution series, 100 μL was vortexed for 10 s and then spread on *Salmonella*-selective agar (*Salmonella* ChromoSelect agar, Sigma Aldrich, GB). The agar plates were incubated at 37 °C for 24 h ± 1 h, after which typical black colonies were enumerated as presumptive *Salmonella* spp. The detection limit was established as 20 colony-forming units (CFU) per gram of compost. Subsequently, one loopful of the sample was spread onto nonspecific agar (Tryptone soya agar, Sigma Aldrich, GB) for MALDI sample preparation.

Detection of *E. coli* was performed using a dilution series in accordance with Czech legislation. 10 g of compost was suspended in 90 mL of 0.9% sodium chloride solution (10^–1^ dilution) and agitated at 150 rpm for 1 h at 4 °C. Triplicates of each dilution were used in a series of cultivation steps as detailed above.

One milliliter of each dilution was added to 9 mL of MacConkey broth (Sigma Aldrich, GB), followed by vortexing for 10 s, and then incubated at 37 °C for 24 h ± 1 h. Then, 100 μL of this enrichment was spread on *E. coli* medium plates (LabMediaServis, Czech Republic) and incubated at 42 °C for 24 h ± 1 h. The detection limit was set at 20 CFU g^−1^. Suspected positive colonies were then transferred to TSA agar for subsequent MALDI verification.

The same procedure as described above for *E. coli* was used to analyze the presence of the indicator microorganism *Enterococcus* spp. The identical dilution series used for *E. coli* was placed directly (100 µL) onto bile esculin azide agar (Merck, Germany) and incubated at 37 °C for 48 h.

Both *Salmonella* spp. and *E. coli* results were documented indicating the confirmed positive (+) or negative (−) results for each of the triplicates at each dilution step.

#### Bacterial isolation—selective agar plate test

Bacterial isolates were obtained from all composting devices. Samples from the bottom and the top of each device were collected, mixed, and used for isolation. Samples were diluted 1:10 (w/v) in 0.9% sodium chloride solution and shaken for 20 min at 30 °C and 150 rpm. Afterward, a tenfold dilution series was prepared. 100 μL of each dilution (10^–2^–10^–7^) was plated in triplicates on selective media to identify the presence of potentially pathogenic bacterial strains while also describing the microbial representation of the compost.

Pseudomonas agar (PSEUSM, BioVendor, Czech Republic), mannitol salt agar (MSA, Sigma Aldrich, GB), kanamycin esculin azide agar (KEA, Merck, Germany), Tergitol T agar (T-7, Merck, Germany), and cetrimide agar (CETR, Merck, Germany) were used in the selective agar plate test to investigate the presence of the commonly occurring and potentially pathogenic microorganisms in the matured products of the composting process. Spore-forming bacteria were isolated on plate count agar (PCA, Oxoid) to demonstrate the presence of endospores in the waste material. The preparation of dilution series followed the same procedure as described for *E. coli*.

Plates were incubated overnight at 37 °C. Colonies were classified based on morphology. For further testing, isolates were cultured on tryptic soy agar (TSA, Sigma Aldrich, GB) and incubated for 48 h at 30 °C. Gram staining was performed to observe the cell morphology and Gram nature of the microorganisms. The methods used in the Gram stain followed those of Madigan (2004).

### DNA extraction and PCR amplification

DNA extraction of bacteria was performed using the myCROBE/croBEE 2.0 Universal Extraction Kit (GeneProof, Czech Republic) in combination with the croBEE® 2.0 Nucleic Acid Extraction System (GeneProof, Czech Republic) according to a previously published procedure (Vršanská et al. 2024).

### Sequence analysis

Sequence analysis was performed using the BigDye® Terminator v3.1 Cycle Sequencing Kit (AB Applied Biosystems, USA) as described by Vršanská et al. (2024).

### Antibiotic susceptibility testing

Antibiotic susceptibility testing was performed using the disk diffusion method according to EUCAST recommendations (EUCAST 2026) as previously described by Vršanská et al. (2024). Antibiotic susceptibility was evaluated using the disk diffusion method with the following antibiotics: ampicillin, ciprofloxacin, tetracycline, gentamicin, erythromycin, vancomycin, and chloramphenicol, following EUCAST 2026 guidelines.

### MALDI-TOF-MS

Representative individual colonies were picked from each agar plate and were identified using the matrix-assisted laser desorption/ionization time-of-flight mass spectrometer (MALDI-TOF MS) Bruker ultrafleXtreme (Bruker Daltonik GmbH, Germany). The identification was performed with the MALDI BioTyper™ Compass Explorer 4.1.90 analysis software, equipped with MBT 8468 MPS library. Individual colonies were either plated directly spread as a thin film, or the protein bacterial isolates were obtained by extraction with ethanol and formic acid and then were pipetted onto the steel MALDI plate. Next, 1 µL of 20 mg/L 2,5-dihydroxybenzoic acid (DHB) matrix in acetonitrile/H_2_O/TFA (50:47.5:2.5) was applied to the same spot and after cocrystallization; the MALDI plate was inserted into the mass spectrometer. Prior to MALDI-TOF identification, an external calibration was performed using a commercial Bacterial Test Standard (Bruker, Germany).

### Statistical analysis

All physicochemical and microbiological parameters were analyzed across three biological replicates per device. Data normality was assessed using the Shapiro–Wilk test. Because several variables did not meet normality assumptions, differences among devices were evaluated using the Kruskal–Wallis nonparametric test with post hoc pairwise comparisons. Results are reported with 95% confidence intervals and replicate variability.

Experimentally measured parameters included temperature profiles, pH, EC, C/N ratio, FTIR spectra, phytotoxicity, and microbiological indicators. Operational characteristics (odor, maintenance, leachate formation, user suitability) were evaluated descriptively as contextual, nonexperimental observations.

## Results and discussion

The differences observed among the four composting systems highlight the importance of matching technological design with intended use, as compost maturity, process duration, and microbiological quality are strongly device dependent. All measured physicochemical and microbiological parameters were statistically evaluated across three biological replicates per device. Because several datasets did not meet normality assumptions (Shapiro–Wilk), differences among composting systems were tested using the Kruskal–Wallis non-parametric test with post-hoc pairwise comparisons. Replicate variability and 95% confidence intervals were included in the evaluation to ensure that observed differences among devices represent consistent trends rather than single‑batch variability.

The input raw material for composting consisted primarily of food waste (FW) collected from the canteen of Mendel University in Brno, supplemented with household FW. A total of 98 kg of FW was used across all devices (GG-02 5 kg, JORA 34 kg, Lifetime 54 kg, Millen 5 kg). The listed FW amounts represent the total mass processed during the full composting cycle in each device, not their daily or maximum loading capacity. The lower quantities processed in the electric units (GG‑02 and Millen) reflect their smaller chamber volumes, which limit batch size and require more frequent loading compared with the larger non-electric systems (JORA and Lifetime). This distinction is important for interpreting process duration and composting performance across devices. The waste included boiled rice, boiled potatoes prepared in various ways, and fresh or processed vegetables (e.g., salads) and smaller amount domestic food waste (bread, meat, dairy). The FW was used without grinding or size reduction. Bulking agents were added only in some devices—GG-02 and JORA used sawdust (1.25 kg and 7 kg respectively), Lifetime used 4 kg of sawdust, while Millen operated without additives or enzymes. All details of experimental design are summarized in Tables 1 and 2 of the main manuscript.

**Table 1:** Characteristics of the composting process

Measurement/composting device	Device GG-02	Device JORA	Device lifetime	Device millen
Amount of FW (kg)	5.00	34.00	54.00	5.00
Additive sawdust (kg)	1.25	7.00	4.00	-
Starting substrate	Yes	No	No	Yes
Measuring of pH during the process	Yes	Yes	Yes	Yes
Measuring of temperature during the process	Yes	Yes	Yes	Yes
Material mixing during the process	Automatic/vertical	Manual/horizontal	Manual/horizontal	Automatic/horizontal
Crushing blades	Yes	No	No	Yes

**Table 2:** Technological process of individual devices during experimental procedure

Device	GG-02	JORA	Lifetime	Millen
Odor	None	Yes	Yes	None
Maintenance	None	Yes	Yes	None
Occurrence of insect	None	Yes	Yes	None
Leachate	None	Yes	Yes	None
Final product	Compost	Compost	Compost	Compost
Number of days in a device	28	103	64	83

### Characterization of technological processes

Four composting devices (GG-02, Lifetime, JORA, and Millen) were compared in terms of operational principles, performance, and suitability for household or small-scale food waste management. Both manufacturer data (Supplementary Material S1) and experimental data are included (Tables 1 and 2)). According to manufacturer information (Supplementary Material S1), the GG-02 and Millen units are fully automatic electric composters with rapid decomposition (24 h) and volume reduction up to 90%. Both devices are able to process a multiplicatively smaller amount of FW than the electricity-independent devices Lifetime and JORA, which require physical involvement in the form of the composting process. Both devices achieve sanitization by heating above 70 °C; however, the Millen requires prehomogenization of waste (≤ 2 cm) and post-treatment maturation. In contrast, Lifetime and JORA composters operate as manual rotary systems without electricity. Composting time ranges from 4 to 12 weeks for Lifetime and 6–8 weeks for JORA, with the latter being insulated, allowing operation at elevated temperatures (up to 75 °C) and improving hygienization efficiency. Although insulated rotary systems such as JORA are capable of reaching elevated temperatures under optimal conditions, the actual temperature profile depends strongly on feedstock composition, moisture content, batch size, and ambient climatic conditions. In this study, the high water content of the food waste (rice, potatoes, vegetables) and the relatively small batch volumes limited heat accumulation, resulting in peak temperatures below 60 °C. These real‑world operating conditions explain the discrepancy between manufacturer‑reported maximum temperatures and the values observed in our experiment. While GG-02 and JORA can process a wider range of food waste including meat and cooked residues, the Lifetime device is limited to plant-based materials. Maintenance is required only for the manual systems.

If composting is to take place indoors, where the odor generated during the composting process is undesirable, the only choice left is between the electrically powered units. The main parameters that can influence the choice of a composting device are listed in Table 2 and Supplementary Material (S1). The electrical composters are equipped with shredding blades and built-in automatic mixing to speed up the composting process. In these composting devices (GG-02 and Millen), the appearance of insects and the formation of infusions were not observed during composting, which makes these devices more indoor user-friendly. In the electric composters (GG‑02 and Millen), no leachate formation was observed. This can be attributed to their operational design, which maintains elevated temperatures and continuous aeration throughout the process. These conditions promote moisture evaporation and prevent the accumulation of free water inside the chamber. At the same time, composter GG-02 was operated with a designated drainage/vent connection that allowed for the removal of water vapor and possibly condensate generated during the cleaning process. In contrast, the non-electric rotary systems (JORA and Lifetime) rely solely on passive aeration, which is less effective at removing excess moisture. As a result, water released during the degradation of food waste can accumulate and form leachate, particularly when the feedstock has a high initial moisture content. Due to their larger chamber volume and longer retention capacity, the Lifetime and JORA devices can accommodate higher food waste inputs compared with the electric units. This makes them more appropriate for households that generate larger quantities of biodegradable waste; however, their suitability ultimately depends on household‑specific waste generation rates and local operating conditions. Automatic devices provide faster processing and lower labor demand at the cost of higher energy consumption and price.

### Temperature

**Fig. 2:**
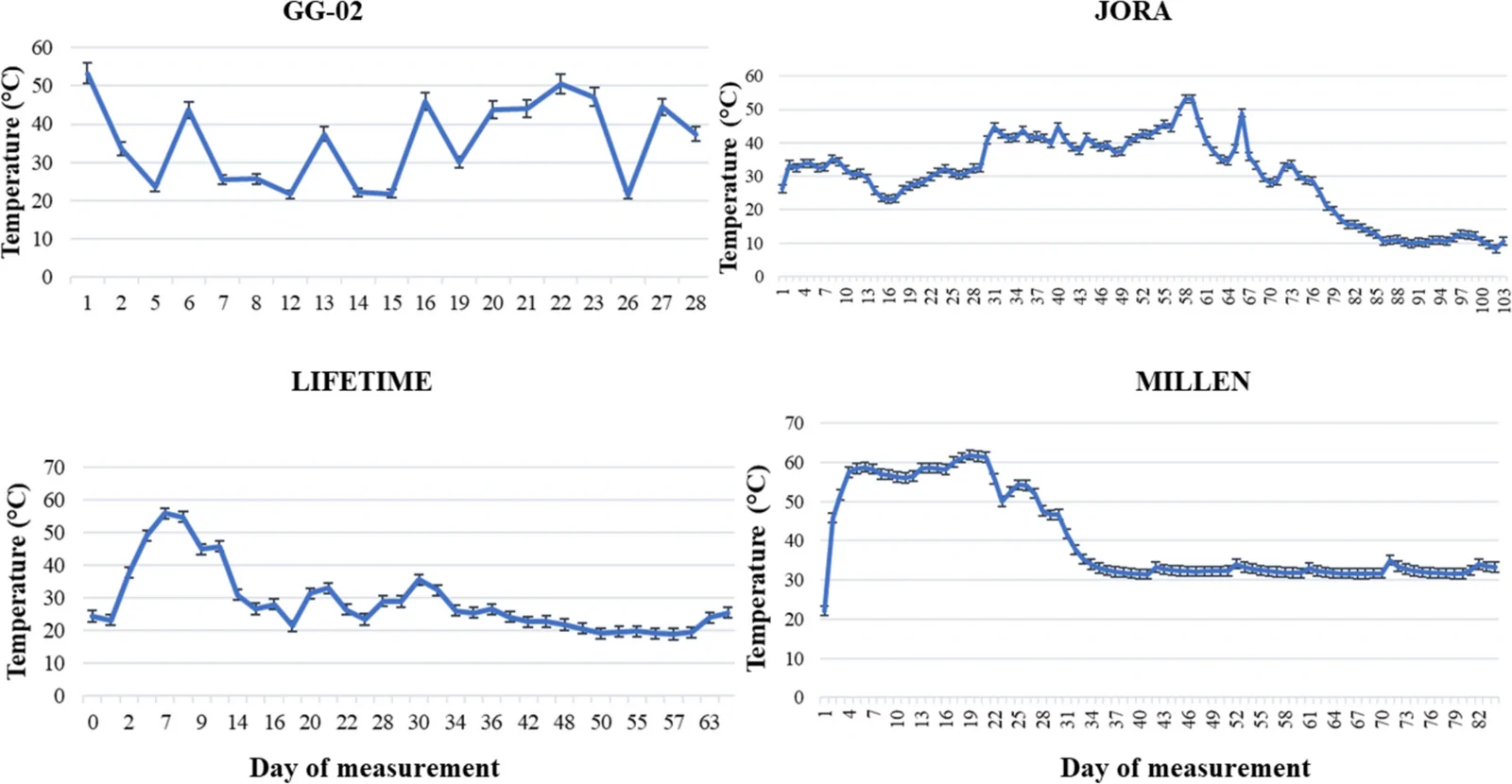
Temperature profile during the composting process in different devices

The temperature was recorded throughout the whole composting process (Fig. 2). The observed temperature profiles suggest that devices with enhanced insulation maintained higher core temperatures for longer durations, potentially improving hygienization outcomes. Given the importance of temperature in composting dynamics, the lack of standardized insulation measures among the four systems presents a limitation in comparing their sanitization efficiency. The results of temperature measurements show that none of the composting devices exceeded 60 °C during operation (Fig. 2). Although insulated or electric units are capable of reaching higher temperatures under optimal conditions, the actual temperature profile depends strongly on feedstock characteristics, moisture content, batch size, and ambient climatic conditions. In this study, the high water content of the food waste (rice, potatoes, vegetables) and the relatively small batch volumes limited heat accumulation, resulting in peak temperatures below the thermophilic threshold. These real‑world operating conditions explain the discrepancy between manufacturer‑reported maximum temperatures and the values observed in our experiment. In the majority of the devices, the temperature profile was observed with a slow tendency to increase and decrease, but in the device GG-02, the temperature was constantly decreasing and increasing; temperature oscillations prevented the establishment of a stable thermophilic plateau. In the GG‑02 device, the observed temperature oscillation resulted from the automatic heating–cooling regulation system, which periodically activates heating elements and mixing cycles to prevent overheating of the small processing chamber. Because the device handles small, high‑moisture batches, the accumulated heat dissipates quickly, leading to repeated warming and cooling cycles rather than a stable thermophilic plateau. Ambient household temperature also contributes to this pattern, as the unit is not fully insulated. These fluctuations limit the establishment of sustained thermophilic conditions and can slow microbial activity compared with systems that maintain more stable internal temperatures. In contrast to the GG-02 device, the Lifetime and JORA devices show a high and slow temperature decrease until the mesophilic phase was reached. Unlike the Lifetime device, the JORA device is insulated, allowing it to reach and maintain higher temperatures for a longer period. In the case of the electric composter, the temperature was controlled automatically. The duration of temperature monitoring reflects the operational composting cycle of each device. Temperature was recorded throughout the entire period during which the material remained inside the composter until removal of the final product. Because the GG‑02 unit has the shortest processing time (28 days), its temperature profile is correspondingly shorter than those of the nonelectric devices, which operate for 64–103 days. No additional post‑processing maturation was included in the temperature measurements, so the profiles shown in Fig. 2 directly represent the active in‑device composting phase.

### pH, EC

**Fig. 3:**
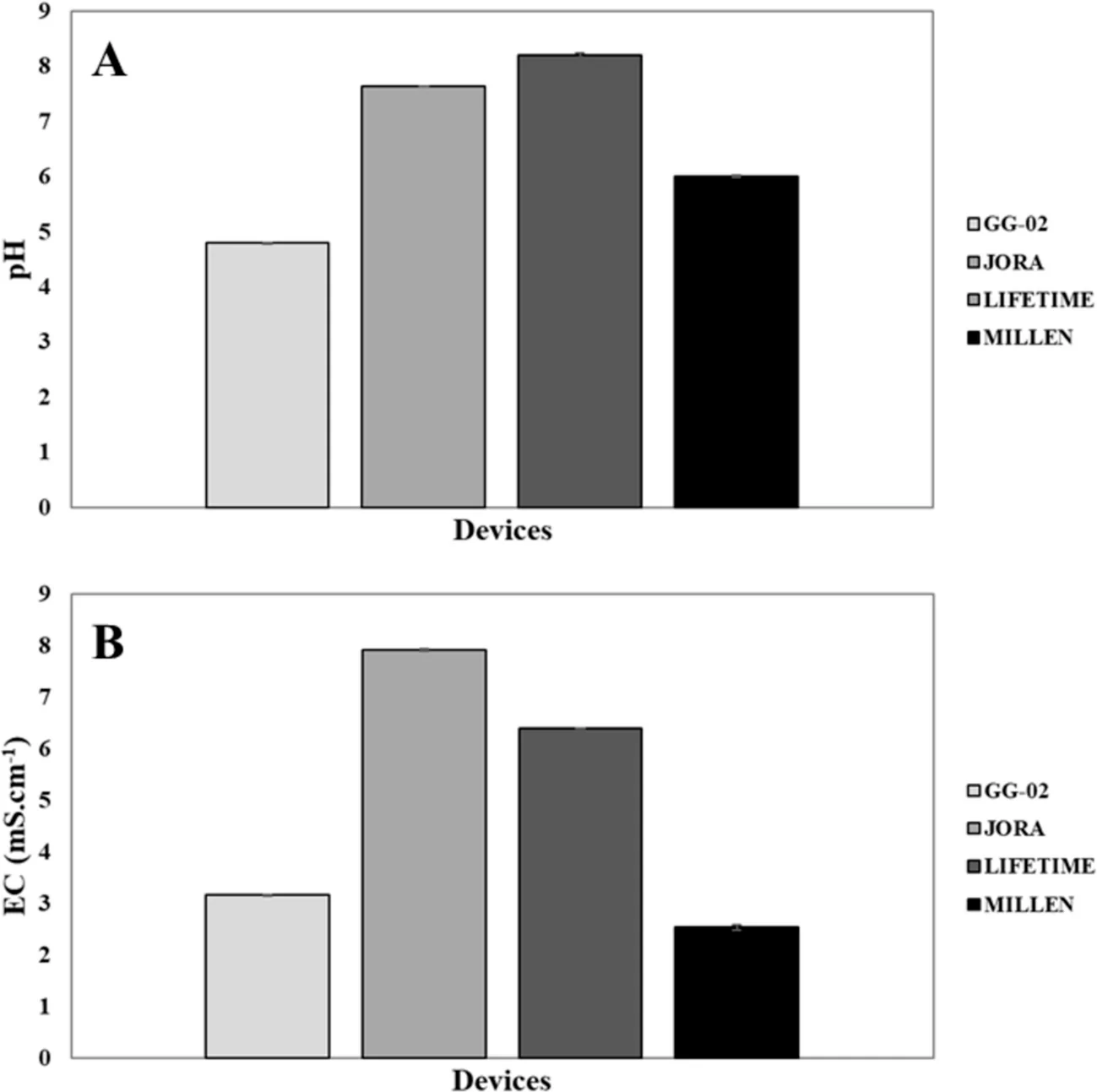
**A** pH and **B** electrical conductivity profile in different composting devices at the end of the composting process

Although pH and EC are not direct indicators of the composting process, they can be used to characterize the resulting products, especially in terms of toxicity. The pH of compost produced by the Lifetime and JORA devices was higher than the pH of compost produced by the GG-02 and Millen devices. The requirements for compost quality (ČSN 2020) are for pH values between 6.0 and 8.5 and were therefore met in all devices, except in the GG-02 device. GG-02 device produced a very acidic compost with a pH value of around 4.5 (Fig. 3A). Acidity is an indicator of immature compost while mature composts have a pH between 7.0 and 9.0 (Tibu et al. 2019), which was observed in the case of JORA and Lifetime devices. Low pH is consistent with conditions that may arise under limited aeration, high moisture content, or rapid acidification of readily degradable substrates; however, oxygen concentration was not measured. The greatest need for aeration of maturing compost is during the hydrolytic phase of maturing, which cannot be provided by fully automated equipment. The difference in pH between the two electric devices reflects their distinct aeration and mixing designs. The Millen composter performs continuous horizontal agitation and allows more effective airflow through the chamber, which reduces the formation of anaerobic microenvironments and supports ammonia release during mineralization. In contrast, the GG‑02 unit relies on intermittent vertical mixing and has a more enclosed chamber, which can trap moisture and create short anaerobic phases that promote organic acid accumulation. Millen also processes smaller, more homogenized batches, improving oxygen penetration and resulting in a higher final pH compared with GG‑02. Differences in pH among devices were statistically significant (Kruskal–Wallis, *p* < 0.05), confirming consistent trends across biological replicates. As the compost matures, the oxidation of the waste in combination with the formation of ammonia changes the pH to an alkaline range (Agapiou et al. 2020), which was not indicated in the case of the GG-02 and Millen devices.

As an indirect indicator of ion concentration in the FW, EC provides information on compost mineralization (Sánchez et al. 2017) and also indicates possible phytotoxic or phytoinhibitory effects (Zhang et al. 2016). Conductivity is related to the concentration of total soluble, dissolved, or suspended salts in the medium. High levels of salinity can be toxic to some sensitive plants (Boeykens et al. 2015). The acceptable level of soluble salts depends on the proposed use of the compost. In this study, the JORA device showed the highest EC values with a value of 8.0 mS·cm^−1^ (Fig. 3B). Madrid et al. (2000) recommended extending the composting time if EC is higher than 5.5 mS·cm^−1^ to maintain a low concentration of salts and to prevent phytotoxicity. Although the Millen composter remained in operation longer than the Lifetime device, its final EC value was lower. This difference reflects the combined effect of feedstock load, mineralization intensity, and moisture dynamics rather than composting time alone. The Lifetime device processed a larger mass of food waste and bulking agent, which promoted more extensive organic matter degradation and led to a higher concentration of soluble salts in the compost. In contrast, the Millen unit handled smaller, more homogenized batches and operated with stronger moisture evaporation, limiting salt accumulation and resulting in lower EC despite the longer in‑device period. In several studies, EC correlated with pH values (Zhang et al. 2016), which was corroborated in our experiments in the case of devices GG-02 and Millen. The higher EC values in the case of composts from JORA and Lifetime devices could be explained by the release of mineral salts consequent to organic matter decomposition (Gómez-Brandón et al. 2008). This is important from an agricultural point of view since a higher EC value is a direct consequence of the increased concentration of nutrients (e.g. nitrates), which can be corrected by adjusting fertilization practices (Sánchez-Monedero et al. 2001). EC values also differed significantly among devices (Kruskal–Wallis, *p* < 0.05), with JORA showing consistently higher conductivity across replicates.

### C/N ratio

**Fig. 4:**
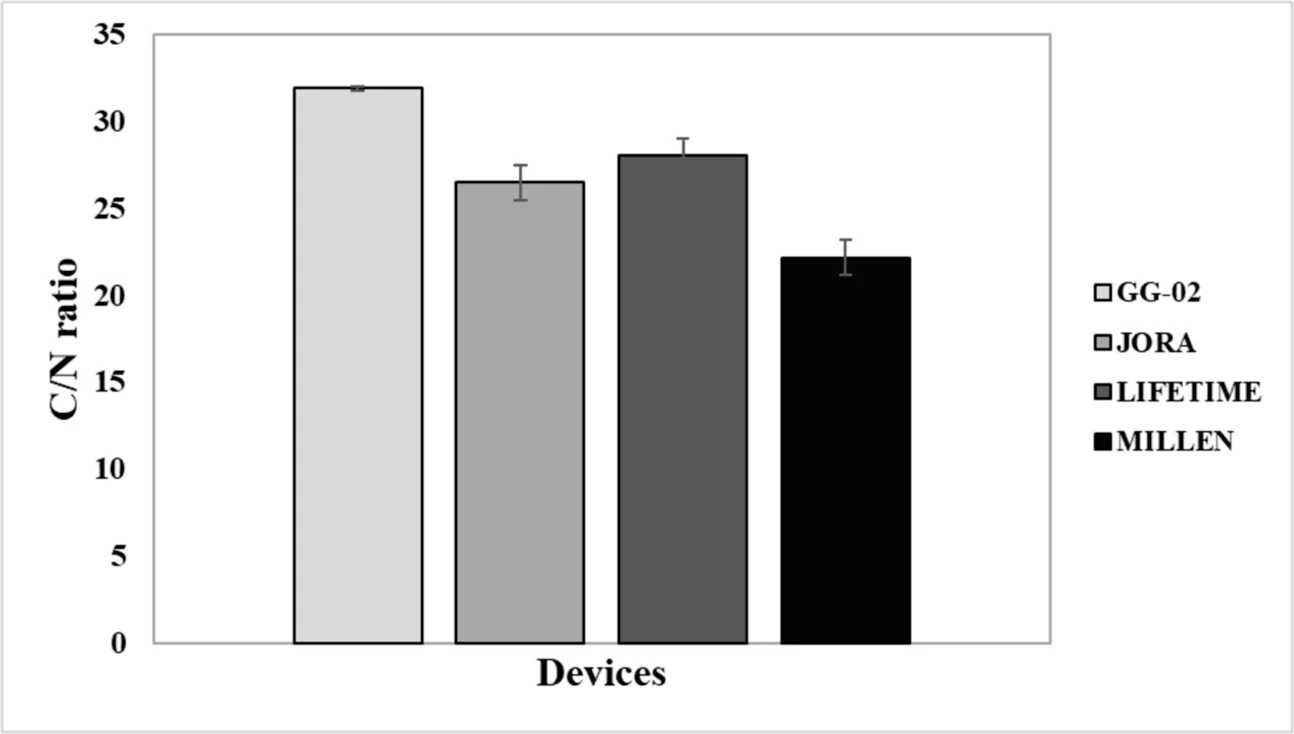
C/N ratio in different composting devices at the end of the composting process

Ensuring a correct carbon to nitrogen (C/N) ratio with a suitable composition of the raw material to be composted is essential to create optimal conditions for the growth of MO. The C/N ratio should be in the range of 30–35 for fresh compost and 25–30 for mature compost (Rajlakshmi et al. 2023) (Fig. 4). The Millen device does not meet these values, where the C/N ratio of mature compost is lower, specifically 22. If the C/N ratio in fresh compost is too tight, the nitrogen content exceeds the metabolic transformation of MO, conversion of ammoniacal nitrogen occurs, and the productivity of humus formation decreases. On the contrary, the GG-02 composter shows a higher value of 32, which can lead to a prolongation of the maturing time of the final compost. Variation in C/N ratios among devices was statistically significant (Kruskal–Wallis, *p* < 0.05), reflecting device‑specific mineralization dynamics.

The superior compost maturity achieved in the JORA and Lifetime systems suggests that lower mechanization and longer retention time can foster more complete organic matter stabilization, supporting previous research on the benefits of slower, aeration‑driven composting processes.

Although the Millen device operated for a longer retention period than the Lifetime composter, its internal process conditions were less favorable for achieving full stabilization. Millen processed smaller, high‑moisture batches and exhibited limited aeration and lower peak temperatures, which slowed mineralization and reduced the degree of humification. In contrast, the Lifetime system handled larger feedstock volumes with more effective passive aeration and more stable temperature dynamics, enabling more complete organic matter decomposition. These results demonstrate that retention time alone does not determine compost maturity; adequate aeration, moisture removal, and thermal conditions are equally essential.

### Humic acids measured by infrared spectroscopy

Infrared (IR) spectroscopy provides appropriate information about the structure and quality of organic matter. The infrared spectra of all the tested composts are similar and differ only in the intensity signals. This indicates that these materials have similar structures but different concentrations of functional groups. The IR spectra of humic acids in composting devices are similar, indicating that different devices had little influence on the types of functional groups of humic acids as the raw material of FW remained the same. However, small changes can be observed.

**Fig. 5:**
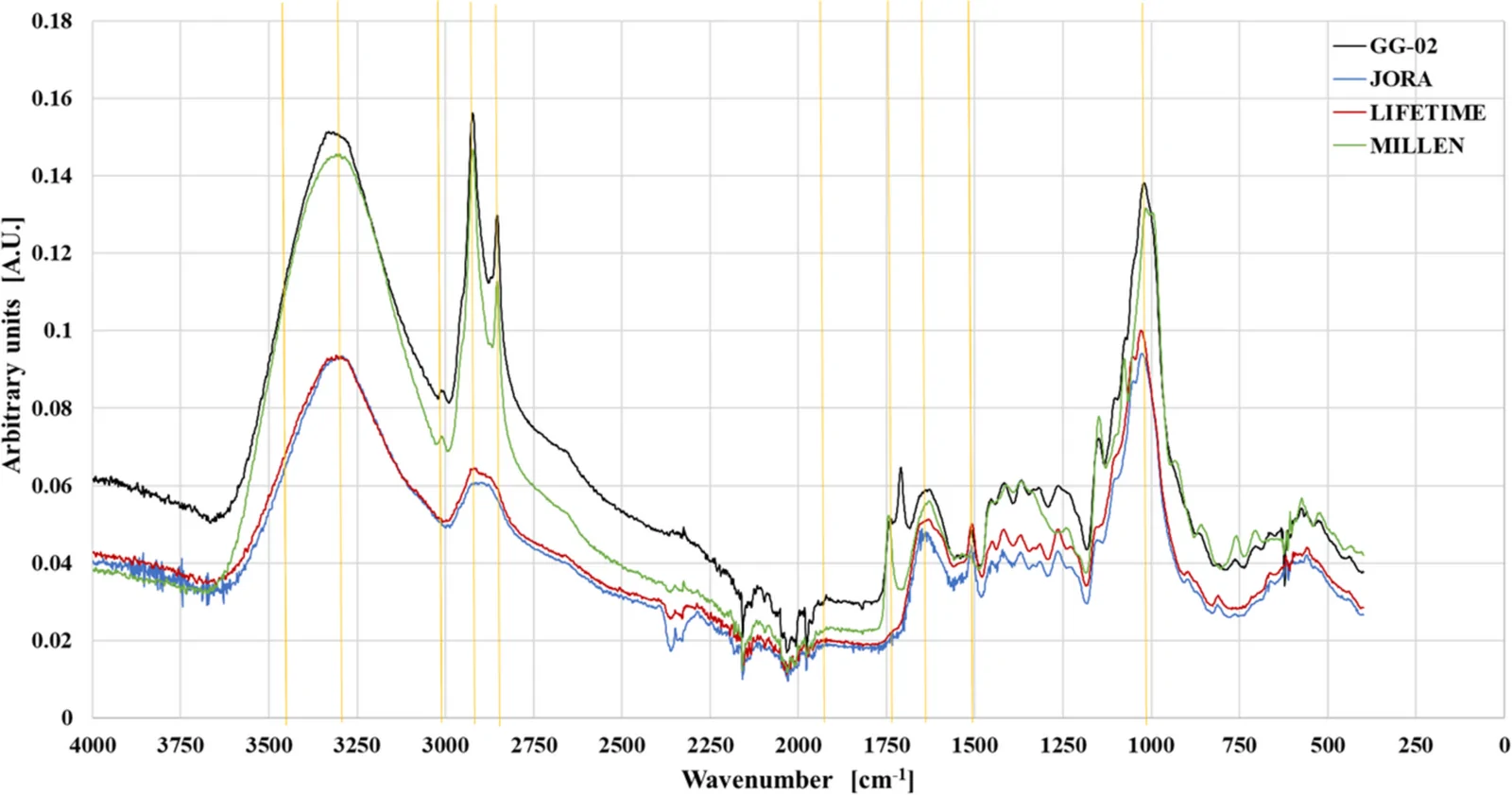
FTIR spectra of FW produced by four types of composter devices

The absorption band at wavelengths around 3030 cm^–1^ observed in devices GG-02 and Millen belongs to valence vibrations of aromatic bonds C–H (Fig. 5). The absence of the absorption band at the wavelength 3030 cm ^–1^ in the spectra of humic acids, as it is in the case of JORA and Lifetime devices, can be caused by practically complete substitution on the aromatic ring or by the overlapping with the adjacent strong band of O–H vibrations in the carboxyl groups which may be extended up to the wavelength 2900 cm ^– 1^ due to hydrogen bonds. Nevertheless, the absence of the absorption band may also be evoked by the interference with the absorption band of water. The absorption band at 2900 cm^–1^, belonging to the valence vibrations of aliphatic C–H bonds, usually has a very variable medium or small intensity in the spectra of humic acids. The antisymmetric and symmetric valence C–H vibration bands in the –CH_2_– groups have maxima at the wavelengths 2930 cm^–1^ and 2840 cm^–1^. According to Salvino et al. (2021), the peak of the aliphatic group area at 2930 cm^−1^ and 2840 cm^−1^ gradually decreased in the case of Lifetime and JORA can be caused by the decomposition of composts by MO, when microbes use the aliphatic and peptide structures and improve the humification process. The absence of bands at the wavelength 1735 cm^–1^, as it is in the case of GG-02, JORA, and Lifetime devices, is considered evidence of only a minimal number of C = O groups being present or that none are bound in saturated esters.

Separate bands with maxima at 1550–1540 cm^–1^, which are characteristic of the valence vibrations of amide, were detected in all the samples from all four devices. This band corresponds to the deformation vibrations of the N–H polypeptides. This band proves the presence of polypeptides in the humic acid macromolecule. A broad and intense absorption band of the valence vibrations –C–OH of aliphatic alcohols has a maximum at wavelength 1150–1050 cm^–1^. In the spectra of ashless humic acids, two or three bands are usually distinguishable at these wavelengths. The most intense one, with a maximum at 1050 cm^–1^ apparently belongs to the O–H vibrations of primary alcohols, which was observed for all four composts.

### Phytotoxicity

The phytotoxicity test focuses on the early development of higher plants and measures the inhibition of seed growth. The higher the percentage calculated, the more toxic the sample is and thus more inhibits seed germination and growth. A 100% share of compost showed a very high toxicity. Compost levels of 1:2 (50%) and 1:4 (25%) showed a decreasing trend in inhibition. The best results were achieved with compost samples from the Lifetime and JORA devices, where seed germination was visible even at 100% share of compost (Table 3).

**Table 3:** Phytotoxicity test

Phytotoxicity compared to OECD soil^a^			
Share of compost/reference soil	1/1	1/2	¼
Samples	100%	50%	25%
GG-02	99.41	96.68	92.12
JORA	98.85	94.13	79.74
Lifetime	98.36	94.49	87.70
Millen	100.00	100.00	99.77
Samples	100% compost	50% compost and 50% reference soil^b^	25% compost and 75% reference soil

^a^OECD soil—OECD soil is a reference substrate supplied in the phytotoxkit. Its use is recommended as an ecotoxicological test substrate to ensure performed tests are intercomparable and repeatable (González Linares et al. 2020). Its composition is 75% air-dried sand, 20% kaolin clay, and 5% finely ground sphagnum peat

^b^Reference soil—natural universal substrate made of a mixture of peat, natural clay, and organic fertilizers was used as reference soil

Phytotoxicity is an important consideration when deciding if and when to apply compost to the soil as a soil amendment. The application of immature compost to the soil has been shown to negatively affect both the early development of seeds and the environment in which it is applied (Cui et al. 2017). The phytotoxicity of immature compost retards seed germination and subsequent growth; it also promotes increased microbial activity in the soil, depleting soil oxygen and blocking available nitrogen is blocked. Phytotoxic components of immature compost include heavy metals, phenolic compounds, and acetic acid (Siles-Castellano et al. 2020).

When evaluating the compost quality, there are two essential parameters, namely, maturity and stability of compost. The maturity of compost indicates a level of its phytotoxic effect on plant development and growth. The stability of compost depends on the content of organic substances resistant to microbial decomposition. Both terms are closely related, as stable compost does not contain biodegradable materials with phytotoxic effects (Oviedo-Ocaña et al. 2015). It is evident from Table 3 that none of the compost produced in any of the composting devices is suitable for application to soil. A positive trend can be seen in the final products of Lifetime and JORA composting devices, which showed a more marked decrease when being applied to reference soil in lower share (25%); however, the phytotoxicity is still very high, which might be attributed to insufficient time provided to the composts to mature which matches with several studies (Bernal et al. 2009; Cui et al. 2017) where fresh composts were found to be highly phytotoxic.

The high phytotoxicity observed across all composts underscores the necessity of extended curing periods before soil application, indicating that household composters—regardless of technology—may require additional maturation steps to ensure agronomic safety. Previous studies on small‑scale and household composting (Voběrková et al. 2020; Ji et al. 2023; Enebe and Erasmus 2023) report that curing periods of several weeks to a few months are typically required after the active composting phase to reduce phytotoxicity and stabilize organic matter before soil application. In the context of Central European household conditions, the composts produced in this study should therefore be regarded as semi-stabilized materials that require additional maturation in garden heaps, beds, or dedicated curing areas to reach agronomically safe quality. From a circular economy perspective, the tested devices function primarily as pretreatment units that divert food waste from mixed municipal streams and initiate its biological transformation. Their suitability as drivers of a local circular economy depends on the availability of space and time for subsequent curing, as well as on users’ willingness to integrate these additional maturation steps into routine household practice.

### Environmental impact assessment

The environmental performance of the tested composting systems varies significantly due to differences in their technological design, energy demand, and compost maturity. Electrically powered units (GG‑02 and Millen) require continuous or frequent electricity input to operate heating, drying, and automated mixing cycles. This increases their indirect greenhouse gas (GHG) emissions depending on the carbon intensity of the local electricity grid. In contrast, the manually operated systems (JORA and Lifetime) do not require electricity, making them inherently less carbon‑intensive during operation and more environmentally favorable from an energy‑use perspective.

Although electric composters automate mixing and heating and therefore shorten the active mechanical processing phase, our results show that they do not necessarily achieve faster overall compost stabilization. Both electric units produced material that still required extended curing, and the Millen device exhibited a longer operational cycle than the Lifetime composter. The higher maturity achieved in the manual systems indicates that process conditions—particularly aeration efficiency, moisture removal, and temperature stability—are more influential than mechanization alone. Consequently, the climate benefits of electric composters depend not only on energy consumption but also on the need for additional maturation steps to obtain agronomically safe compost. A simplified comparison suggests that electrically driven systems may consume several kilowatt‑hours per batch, while non-electric systems operate at nearly zero operational energy cost. Therefore, the environmental trade‑off lies between faster processing with higher energy consumption and longer processing with minimal environmental burden.

In addition to energy use, the environmental risk associated with compost maturity must be considered. Immature compost with low pH and elevated electrical conductivity can cause soil acidification, osmotic stress in plants, and leaching of soluble salts into groundwater. The presence of phytotoxic compounds—such as volatile organic acids and phenolic compounds—in immature compost represents a further environmental hazard by inhibiting seed germination and negatively affecting soil microbial communities. Because all compost samples demonstrated high phytotoxicity at 100% concentration, direct land application would pose environmental risks and should be avoided without extended curing.

Leachate formation, especially in outdoor and non-insulated devices, may also contribute an environmental burden. Nutrient‑rich liquids can carry dissolved organic carbon, nitrogen, and mineral salts that, if improperly managed, may contaminate wastewater systems or surface waters. Although the electric composters did not produce measurable leachate, their high energy consumption presents a different form of environmental cost.

From a circular economy perspective, all four composting systems promote nutrient recovery and reduce municipal waste streams; however, their overall sustainability must be interpreted through a life-cycle lens. When applying life-cycle thinking, nonelectric systems appear to offer lower environmental burdens per kilogram of processed waste due to their minimal resource use and production of more mature compost. Electric systems, while more energy‑intensive, provide better hygienization, odor control, and indoor usability. These contrasting profiles suggest that environmental decision‑making should consider both direct impacts (energy, emissions, compost quality) and indirect impacts (usability, avoidance of landfill methane, and suitability of the compost for soil application).

Overall, JORA and Lifetime show a more favorable environmental profile due to low energy use, more stable compost, and reduced need for post‑processing. In contrast, GG‑02 and Millen minimize odor and insect occurrence but require additional maturation time and exhibit higher indirect emissions linked to electricity consumption. These trade‑offs indicate that environmentally optimal selection of household composting technology depends on balancing energy inputs with compost quality and intended end‑use.

### Economic assessment

An economic comparison of the composting systems provides additional context for evaluating their practical applicability in household settings. Electrically powered devices (GG-02 and Millen) incur operating costs associated with electricity consumption, whereas manually operated systems (JORA and Lifetime) require only user labor and routine mechanical maintenance. Although electric composters offer faster processing and improved indoor usability, these advantages come at higher operational expenses.

#### Operational energy use and estimated running costs

Based on typical manufacturer specifications for household electric composters, the average power consumption ranges between 300 and 500 W during active heating and mixing cycles. Assuming an average daily runtime of 1–2 h, the estimated electricity consumption per batch is approximately 0.6–1.0 kWh. In contrast, nonelectric systems have negligible operational energy use, making their running costs effectively zero.

To contextualize these values relative to waste throughput, the approximate energy intensity is 0.10–0.20 kWh per kilogram of food waste processed in electric units, whereas the manual systems operate without energy input. Higher energy demand increases the long-term cost of operation and influences the overall environmental profile of electric composters.

#### Economic feasibility and long-term affordability

From a cost-benefit perspective, nonelectric composters (JORA and Lifetime) offer the lowest operational costs and the highest long-term affordability. Their extended residence time and need for manual handling may be offset by the absence of recurring energy expenses. Electric units provide improved comfort, shortened processing time, and indoor usability; however, these benefits come with ongoing energy costs and potentially higher maintenance needs due to mechanical components.

When considering investment and operating expenses together, the choice between electric and manual systems depends on user priorities: convenience and speed versus cost efficiency and long-term sustainability. Economic considerations are based on manufacturer‑reported purchase prices and qualitative observations; no formal cost modeling or sensitivity analysis was conducted. Household preferences regarding composting technologies have been described in several behavioral and adoption studies, which report that users generating larger quantities of food waste tend to favor low-cost, low-maintenance manual systems, while households prioritizing odor control, convenience, and indoor operation more often adopt automated electric units. These trends reflect general patterns observed in small‑scale composting research rather than primary social data collected in this study. In our context, the performance characteristics of the tested devices align with these previously reported user preferences, suggesting that both manual and electric systems can serve different household needs within a local circular-economy framework.

### Microbiological analyses

Composting of FW is a major concern worldwide due to the potential adverse effects of pathogenic microorganisms or toxic leachates that can reach agricultural soil or water systems. The quality of the final compost derived from FW is an important factor in the potential application of the compost product to the soil. The composting process depends on the microbial community of beneficial microbes with degradation potential.

Evaluation of the microbial composition of the final composting product and identification of the potential common microbial marker of the final product of the composting process has been done. The microbial analysis of samples collected at the end of maturation phase was carried out using several different methods. In the first step, a cultivation method on selective media was used to isolate single bacterial colonies for subsequent identification. The media were selected to differentiate potentially pathogenic and commonly encountered bacterial strains in FW such as *Enterobacter* spp., *Escherichia coli*, *Klebsiella* spp., *Proteus* spp., *Salmonella* spp., and *Shigella* spp. Identification of cultured colonies consisted of identification by Gram staining to differentiate between Gram-positive and Gram-negative bacteria followed by morphological determination.

The highest bacterial load was found in JORA, followed by Lifetime and Millen composter, and the lowest bacteria load was found in GG-02 (Table 4). The reduced microbial loads in electric devices, while positive from a hygiene standpoint, also reveal a potential drawback: the suppression of beneficial microbial activity may delay humification and reduce the formation of stable organic fractions. The study showed that GG-02, due to the automatic cycling of temperature profiles and the elimination of excess moisture, although it successfully sanitizes the compost and prevents the multiplication of undesirable microorganisms, strongly suppresses the natural microbial process of composting. The microbiome of the compost is quickly exposed to adverse conditions such as high temperature and low humidity, which directly negatively affects the occurrence of all commonly occurring microorganisms in all types of compost.

**Table 4:** Results of the selective media panel test

Selective media											
Device sample	TSA	PSEUSM	MSA	PC	BDC	BE	KEA	McC	CETR	HCTM	T-7
	Colony-forming unit (CFU) per 1 g of mature compost derived from FW										
GG-02	0	0	0	0	360	0	0	0	0	0	0
JORA	82,522	21,621	97,297	456,756	379,279	2727	0	0	0	0	0
Lifetime	49,090	49,090	50,122	0	3316	3619	0	0	0	0	0
Millen	53,551	33,353	53,663	63,466	25,335	0	0	0	0	0	0

Cultivations were performed in decadic dilution 10E−1–10E−5. Cultivation on selective media to identify the presence of potentially pathogenic bacterial strains showed positivity on PSEUSM and MSA agars. KEA, T-7, and CETR did not show any colony formation. Based on these results, the final compost derived from FW does not contain any *Enterobacter* spp., *Escherichia coli*, *Klebsiella* spp., *Proteus* spp., *Salmonella* spp., and *Shigella* spp. neither pathogenic species *Pseudomonas aeruginosa*

*TSA* tryptic soya agar, *PSEUSM* Pseudomonas agar, *MSA* mannitol salt agar, *PC* plate count agar, *BDC* BD™ Columbia agar with 5% sheep blood, *BE* bile esculin azide agar, *KEA* kanamycin esculin azide agar, *McC* MacConkey agar, *CETR* cetrimide agar, *HCTM* HiChrome coliform agar, *T-7* Tergitol T agar

Colonies from selective agars were separated and sampled for sequencing analysis and MALDI analysis. In total, 16 different samples were sequenced and identified across the three composting devices JORA, Lifetime, and Millen. No colonies could be isolated and sequenced from the final compost product sampled from the GG-02 composting device.

Independent cultivation of FW-compost on different types of media showed results consistent with the preceding results of cultivation on selective media. The plate count agar was used to compare the total amount of bacteria in different composting devices. The results repeatedly confirm JORA and Lifetime as the composting devices with the highest amount of bacteria in mature compost samples in comparison with Millen and GG-02. The cultivation results on selective agars did not show any colony formation of coliform bacteria *Salmonella* spp. and *E. coli* in any of the composting devices (Table 5). The results confirm the previous cultivation results. The presence of small amounts of *Enterococcus* spp. was detected in JORA and Lifetime. The cultivation allowed the detection of sporulating bacteria in high amounts in the JORA and the Lifetime composting system (Table 4).

**Table 5:** Parameters of final product

	Limits (ČSN 2020)	GG-02	JORA	Lifetime	Millen
Nondegradable parts < 2 cm (%)	< 3	1.023	2.739	2.486	20.833
*Salmonella* sp.	0	0	0	0	0
*E. coli*	4 < 10^3^	0	0	0	0
*Enterococcus*	4 < 10^3^	0	1	3	0

The sequence analysis allowed the identification of bacterial strains, and the results were consistent with the results of independent cultivation. *Sporosarcina koreensis* was identified in the sample cultivated on TSA agar from JORA composting device, where sporulating bacteria were detected by independent cultivation. Sequence analysis also confirmed *Staphylococcus equorum* in JORA samples cultivated on MSA agar (Table 6).

**Table 6:** Results of the isolation from different composting devices: Gram staining and sequencing (morphology results detailed in Supplementary Material S2)

Streak plate method	Sample number	Sequencing	MALDI	Spore-forming bacteria
1A-53 TSA JORA	4	*Bacillus* sp./*licheniformis*	X	+
1A-51	16	*Sporosarcina koreensis*	X	+
1A-52	11	*Bacillus paralicheniformis*/*licheniformis*	*Bacillus licheniformis*	+
1D-41 JORA MSA	6	*Staphylococcus equorum*	*Staphylococcus equorum*	
1D-42	14	*Bacillus licheniformis*	*Bacillus licheniformis*	+
1F-41 JORA PSEU	10	*Bacillus licheniformis*	X	+
3A-51 Lifetime TSA	9	*Lysinibacillus* sp.	*Lysinibacillus massiliensis*	+
3A-52	12	*Bacillus* sp./*licheniformis*	X	
3D-41 Lifetime MSA	7	*Bacillus licheniformis*	*Bacillus licheniformis*	+
3D-42	15	*Bacillus licheniformis*	*Bacillus licheniformis*	+
3F-32 Lifetime PSEUM	8	*Cytobacillus kochii*	X	
4A-41 TSA Millen	5	*Bacillus licheniformis*	*Bacillus licheniformis*	+
4D-41 MSA Millen	3	*Bacillus licheniformis*	X	+
4F-31 PSEUM Millen	2	*Bacillus* sp./*licheniformis*	X	+

*Bacillus licheniformis* was detected in all three composting systems JORA, Lifetime, and Millen in several samples from TSA, MSA, and PSEUSM agar (Table 6). The presence of this species was confirmed by MALDI as the independent method used for bacteria identification. Species *B. licheniformis*, a common soil bacterium, is important for food microbiology because it can cause a food-borne diarrheal illness (Logan 1988). *Lysinibacillus* sp. and *Cytobacillus kochii* were also identified in the Lifetime device. *Lysinibacillus* sp. was also detected by MALDI and identified to the species level as *Lysinibacillus massiliensis*.

*Bacillus licheniformis* is commonly found in FW and is one of the bacteria that have been found to be involved in composting processes. The temperature range of 50 to 65 °C is of selective advantage for bacteria. The *B. licheniformis* is also used as an inoculant to influence organic degradation, enzyme activity, and bacterial community dynamics (Kim et al. 2021; Su et al. 2024; Wang et al. 2024).

Antimicrobial resistance is one of the major threats to public health and according to the World Health Organization (WHO), Geneva, Switzerland, the antibiotic resistance is increasing over time throughout the world (WHO New Report 2019). The FW is a natural reservoir for the distribution of antibiotic resistance strains across the whole bacterial spectrum, so the knowledge of resistance rates is important to determine and potentially prevent the spread of antibiotic resistance (Gajic et al. 2022; Yashwant et al. 2023; Periasamy et al. 2023). Antimicrobial susceptibility testing is commonly used method to determine the antibiotic resistance, and the disk diffusion test remains the most widely used routine antimicrobial susceptibility test in clinical microbiology laboratories (Gajic et al. 2022).

Antibiotic susceptibility testing was performed on *Bacillus licheniformis* found in three composting devices. Ten different isolates of *B. licheniformis* were tested for susceptibility to antibiotics recommended by EUCAST (2026). The isolates were obtained from different composting systems (Table 7).

**Table 7:** Determination of antibiotic sensitivity in selected samples of bacteria within the tested samples of FW

Bacteria	Sample/system/medium	Antimicrobial agent	Disk content (µg)	Zone diameter (mm)	Zone diameter breakpoints (mm) EUCAST S ≥/R <	Disk contents (µg) EUCAST
*Bacillus*						
*Bacillus licheniformis*	1A-53/JORA/TSA	Ciprofloxacin	5	36	50/23	5
		Vancomycin	30	24	10/10	5
		Erythromycin	15	32	24/24	15
		Linezolid	10	34	22/22	10
		Meropenem	10	40	25/25	10
*Bacillus licheniformis*	1A-52/JORA/TSA	Ciprofloxacin	5	36	50/23	5
		Vancomycin	30	24	10/10	5
		Erythromycin	15	28	24/24	15
		Linezolid	10	34	22/22	10
		Meropenem	10	40	25/25	10
*Bacillus licheniformis*	1D-42/JORA/MSA	Ciprofloxacin	5	36	50/23	5
		Vancomycin	30	34	10/10	5
		Erythromycin	15	22	24/24	15
		Linezolid	10	34	22/22	10
		Meropenem	10	30	25/25	10
*Bacillus licheniformis*	1F-41/JORA/PSEU	Ciprofloxacin	5	38	50/23	5
		Vancomycin	30	26	10/10	5
		Erythromycin	15	20	24/24	15
		Linezolid	10	38	22/22	10
		Meropenem	10	44	25/25	10
*Bacillus licheniformis*	3A-52/Lifetime/TSA	Ciprofloxacin	5	40	50/23	5
		Vancomycin	30	26	10/10	5
		Erythromycin	15	26	24/24	15
		Linezolid	10	28	22/22	10
		Meropenem	10	40	25/25	10
*Bacillus licheniformis*	3D-41/Lifetime/MSA	Ciprofloxacin	5	36	50/23	5
		Vancomycin	30	26	10/10	5
		Erythromycin	15	32	24/24	15
		Linezolid	10	36	22/22	10
		Meropenem	10	34	25/25	10
*Bacillus licheniformis*	3D-42/Lifetime/MSA	Ciprofloxacin	5	28	50/23	5
		Vancomycin	30	22	10/10	5
		Erythromycin	15	32	24/24	15
		Linezolid	10	36	22/22	10
		Meropenem	10	38	25/25	10
*Bacillus licheniformis*	4A-41/Millen/TSA	Ciprofloxacin	5	30	50/23	5
		Vancomycin	30	22	10/10	5
		Erythromycin	15	22	24/24	15
		Linezolid	10	36	22/22	10
		Meropenem	10	34	25/25	10
*Bacillus licheniformis*	4D-41/Millen/MSA	Ciprofloxacin	5	32	50/23	5
		Vancomycin	30	20	10/10	5
		Erythromycin	15	35	24/24	15
		Linezolid	10	38	22/22	10
		Meropenem	10	34	25/25	10
*Bacillus licheniformis*	4F-31/Millen/PSEUM	Ciprofloxacin	5	28	50/23	5
		Vancomycin	30	18	10/10	5
		Erythromycin	15	35	24/24	15
		Linezolid	10	28	22/22	10
Others						
*Staphylococcus equorum*		Meropenem	10	28	25/25	10
	1D-41/JORA/MSA	Tetracycline	30	40/45	22/22	30
		Cefoxitin	30	38/36	22/22	30
		Ciprofloxacin	5	40/34	50/22	5

The breakpoints established by the European Committee on Antimicrobial Susceptibility Testing (EUCAST 2026), outputs: *S* susceptible, *I* susceptible, *R* resistant: I category is interpreted as values between the S and the R breakpoints

The antibiotics tested included carbapenems, fluoroquinolones, glycopeptides, macrolides, and oxazolidinones. No antibiotic resistance to multiple types of antibiotics was observed in any of the samples tested. The susceptibility of *Staphylococcus equorum* to several antibiotics was also tested. No resistance was detected, and the isolated bacterial colony was susceptible to all antibiotics tested.

Interpretation of device performance is limited by differences in chamber volume, feedstock mass, moisture content, and retention time, which are inherent to the technologies and represent real‑world operational conditions rather than controlled laboratory standardization.

## Conclusion

This study provides a comparative evaluation of four household composting systems under realistic operating conditions. The results show that device design strongly influences temperature development, physicochemical parameters, and compost maturity. None of the systems produced fully stabilized compost, and all final products exhibited phytotoxicity, indicating the need for an extended curing phase. Microbiological analyses confirmed the absence of *Salmonella* spp. and *Escherichia coli* in all devices, and no antibiotic-resistant strains were detected among the cultured isolates. Differences in feedstock mass, chamber volume, moisture content, and retention time represent inherent operational characteristics of each technology and should be considered when interpreting device performance. Overall, the findings highlight the importance of post-treatment and careful technology selection for safe and effective household food waste composting.

## Supplementary information

Below is the link to the electronic supplementary material.

ESM 1 (DOCX 18.1 KB)

ESM 2 (DOCX 15.0 KB)

## Data Availability

The datasets generated and analyzed during the current study are available from the corresponding author upon reasonable request.
