# Supplementary material for: Comparative evaluation of four household food‑waste composting systems for decentralized circular waste management
Source: Environ Sci Pollut Res Int. 2026 Oct 10;33(35):17086–102. doi: 10.1007/s11356-026-38301-x (PMC13683473; doi:10.1007/s11356-026-38301-x)
Supplement: Supplementary file 1 [file 11356_2026_38301_MOESM1_ESM.docx]

SUPPLEMENTARY INFORMATION

The GG-02 composter (Figure 1a) is suitable for households or smaller catering establishments (schools, offices, companies). Its daily capacity is 5 kg. It is an electric and fully automated composter. The Lifetime rotary composter (Figure 1b) works on the principle of a classic garden composter, with a technological difference of bringing the air inside the device by rotation. It is a hexagonal composter made of durable HDPE plastic and a fixed metal structure. The JORA device (Figure 1c) is a two-chamber rotary composter developed for composting biowaste, and kitchen FW. The inner side of the composter is insulated which enables composting also during the winter months. Millen (Figure 1d) is a device using an automatic system for FW processing. The device is delivered with a starter substrate that contains microorganisms and sawdust. The starter substrate needs to be activated with water and allowed to stand for several hours before use. Only after the activation the FW can be added.

**The GG-02 composter**

Its principle is based on a rapid decomposition of organic substances which is triggered by a unique mixture of starter cultures of microorganisms included in the package. Thanks to the activity of the microorganisms, the volume is reduced by 80-90% in 24 hours. This composter is capable of processing all types of FW, including meat products (small bones, meat scraps, fish bones and mussels) and food that is not suitable for consumption, i.e. food after warranty, coffee grounds, and also toilet paper rolls and animal litter. This composter is made of ABS plastic. The inner container is made of stainless steel. The composter is equipped with a single-chamber system with automatic rotating propellers that mix the input raw materials and break larger parts of the waste into smaller ones. The sanitization process is achieved thanks to the temperature which reaches over 75°C for a period of one hour. This temperature guarantees the elimination of harmful pathogens and ensures the microbiological safety of the final compost.

**The Lifetime rotary composter**

A pipe supplying the air during the rotation and at the same time conducting water and toxic juices produced during the composting process out is installed inside the device. Input raw materials should consist of kitchen and garden waste. The composter is not suitable for processing meat, bones and dairy products. Garden waste, which may contain pesticides and herbicides, plastic films or animal excrement, is neither recommended. Raw materials should be loaded to the composter at once, otherwise the composting process can be disrupted and prolonged. New input raw material should be only loaded after the original material has settled. The composting time ranges from 4 to 12 weeks depending on the input raw materials and the composting conditions. The composting process needs to be controlled which requires physical and manual involvement. Composter should be turned several times a week depending on the progress of composting. In order to ensure a proper composting process, it is necessary to maintain the appropriate humidity of the compost. When the compost reaches a dark-brown colour and has an earthy smell it is possible to finish the composting process. A final product can be applied directly to the soil or used as a mulch. In case the compost should serve as a substrate for vegetable planting, it needs to be allowed to mature for approximately 8 weeks and then it can be incorporated into the soil.

**The JORA device**

Its daily capacity is between 25 to 30 liters of waste. The working principle is similar to the one of the Lifetime device. Thanks to the insulation, a temperature of up to 75°C is reached during the composting process which facilitates the reduction of composting time to around 6 to 8 weeks. This property ensures a higher and safer hygienization and makes this composter suitable for FW composting. Suitable FW includes food scraps (cooked and raw), meat products with bones, vegetables, whole eggs, pastries, paper tissues, fruit and citrus fruits. Other raw materials are considered unsuitable similarly to other devices. It is recommended to add sawdust or pellets to the input loading. The final product is supposed to be compost usable as fertilizer.

**The Millen device**

The minimum waste capacity is 1 kg and the maximum capacity is 5 kg per cycle. The manufacturers guarantee the decomposition of the organic material in 24 hours, however, the raw material needs to be homogenized with a maximum size of a piece 2 cm. Common compostable raw materials such as vegetable raw materials, as well as bread and pastries, leftovers from kitchen meals and meat products are considered suitable materials. Bones, paper and plastic scraps, medicinal pills are unsuitable. After the loading with suitable raw materials, the blades are activated in the device and crush the raw materials into smaller pieces. The device automatically monitors humidity and oxygen content. Other gases are captured using a deodorization system, which is provided by a UV-lamp. After the completion of the composting process, the final product needs to rest for 2 weeks before the application to the soil in a ratio of 5% compost and 95% substrate.

*Table S1: Comparison of composting devices* (abbreviation n.s.m means not specified by the manufacturer). Information was provided by the manufacturer and may differ from the conditions reported in the study.

| **Measurement/composting device** | **Device GG-02** | **Device Lifetime** | **Device JORA** | **Device Millen** |
| --- | --- | --- | --- | --- |
| **Composting time** | 24 hours | 4-12 weeks | 6-8 weeks | 24 hours |
| **Amount of compost (kg/cycle)** | 4 | n.s.m | 25-30 | 5 |
| **Consumption** | Approximately 100 W/h | / | / | 300 W/h |
| **Dimensions – height x width x lenght (cm)** | 76.2 x 45.7 x 45.7 | 109.7 x 89.1 x 90.9 | 125 x 92 x 70 | 67 x 65 x 82 |
| **Volume (l)** | n.s.m | 246 | 270 | n.s.m |
| **Weight (kg)** | 27 | 20.4 | 38 | 110 |
| **Automatic/manual (A/M)** | A | M | M | A |
| **Volume reduction %** | 90 | 90 | 90 | n.s.m |
| **Final product** | Compost | Compost | Compost | Compost |
| **Maintenance** | NO | YES | YES | NO |
| **Material** | **ABS plastic +** **stainless steel** | **HDPE plastic** | **HDPE plastic +**  **stainless steel** | **Stainless steel** |
