# Supplementary material for: Comparative evaluation of four household food‑waste composting systems for decentralized circular waste management
Source: Environ Sci Pollut Res Int. 2026 Oct 10;33(35):17086–102. doi: 10.1007/s11356-026-38301-x (PMC13683473; doi:10.1007/s11356-026-38301-x)
Supplement: Supplementary file 2 [file 11356_2026_38301_MOESM2_ESM.docx]

*Table S5: Detailed morphology results*

| **Streak Plate Method** | **Sample number** | **Gram stain/Gram´s method/ Morphology** | |
| --- | --- | --- | --- |
| **1A-53 TSA JORA** | 4 | G+ / matt, irregular shape, with raised center, wavy edge, 1-2 mm, cream color |  |
| **1A-51** | 16 | G+ rods  glossy, round, flat, continuous edge, 1-2 mm, beige to brown |  |
| **1A-52** | 11 | G+ / glossy, irregular shape, flat, lobed edges, 3-4 mm, cream color |  |
| **1D-41 JORA MSA** | 6 | G+ cocci |  |
| **1D-42** | 14 | G+ / matt, irregular shape, flat, lobed edge, 2 - 4 mm, cream color |  |
| **1F-41 JORA PSEU** | 10 | G+ / matt + glossy edges, irregular shape, flat, lobed irregular edge, 1-2 mm, color cream |  |
| **3A-51 Lifetime TSA** | 9 | G+ / glossy, round, flat, continuous edge, appearance of a "ring" around the colony, 1-2 mm, color cream |  |
| **3A-52** | 12 | G+ / matt, irregular shape, flat, lobed edge, 1-2 mm, cream color |  |
| **3D-41 Lifetime MSA** | 7 | G+ / thickened center, irregular, with raised center, lobed margin, 1 - 2 mm, cream color |  |
| **3D-42** | 15 | G+ / matt, irregular shape, flat, lobed edge, 2 - 4 mm, cream color |  |
| **3F-32 Lifetime PSEUM** | 8 | G+ / matt + glossy edges, irregular, flat, lobed edge, 3-4 mm, cream |  |
| **4A-41 TSA Millen** | 5 | G+ / thickened center, irregular, flat, lobed edge, 1-2 mm, cream color |  |
| **4D-41 MSA Millen** | 3 | G+ / glossy, concentrically striped, raised center, lobed edge, 1-2 mm, cream color |  |
| **4F-31 PSEUM Millen** | 2 | G+ /semi-matt, thickened in the center, concentrically striped, flat, fibrous margin, 1-2 mm, cream color |  |
